# [Retracted] PADI4-mediated epithelial-mesenchymal transition in lung cancer cells

**DOI:** 10.3892/mmr.2025.13610

**Published:** 2025-07-07

**Authors:** Meiyan Liu, Yang Qu, Xue Teng, Ying Xing, Dandan Li, Chunhong Li, Li Cai

Mol Med Rep 19: 3087–3094, 2019; DOI: 10.3892/mmr.2019.9968

Following the publication of this paper, it was drawn to the Editor's attention by a concerned reader that certain of the western blot data shown in Fig. 5C on p. 5092 were strikingly similar to data that had already been published in different form in the journal *Oncotarget* in an article written by different authors. In addition, upon analyzing the data in this paper independently in the Editorial Office, one pair of overlapping data panels was identified comparing the scratch-wound assay data in Fig. 2A with that in Fig. 4A, whereas two pairs of overlapping data panels were identified for the invasion assay data shown in Fig. 4B (thereby affecting all the panels), such that data which were intended to show the results from differently performed experiments had apparently been derived from the same original source.

Owing to the fact that the contentious western blot data mentioned above in Fig. 5C had already apparently been published prior to its submission to *Molecular Medicine Reports*, the Editor has decided that this paper should be retracted from the Journal. After contacting the authors, they accepted the decision to retract the paper. The Editor apologizes to the readership for any inconvenience caused.

